# DNA origami-based virus-like particles: a new class of HIV vaccine candidates with superior immunogenic properties

**DOI:** 10.1038/s41392-026-02735-z

**Published:** 2026-05-22

**Authors:** Frauke Muecksch, Juan Hamdan, Oliver T. Fackler

**Affiliations:** 1 Center for Integrative Infectious Disease Research (CIID), Virology, Heidelberg University, Medical Faculty Heidelberg, Heidelberg, Germany; 2 Chica and Heinz Schaller (CHS) Research Group, Department of Infectious Diseases, Virology, University Hospital Heidelberg, Heidelberg, Germany; 3 German Centre for Infection Research (DZIF), Partner Site Heidelberg, Heidelberg, Germany; 4 SynthImmune Cluster of Excellence, Heidelberg University, Heidelberg, Germany; 5 Center for Integrative Infectious Disease Research (CIID), Integrative Virology, Heidelberg University, Medical Faculty Heidelberg, Heidelberg, Germany

**Keywords:** Vaccines, Vaccines

Research Highlight Romanov et al. 2026 Science;391(6785):eadx6291

In a paper recently published in *Science*, Romanov et al. present a new anti-HIV vaccine prototype using a DNA origami scaffold for antigen delivery that primes and expands rare broadly-neutralizing antibody (bnAb) precursor B cell clones in a humanized mouse model without triggering unwanted anti-scaffold antibody responses.^1^ This study demonstrates the potential of DNA origami as immunogen carrier towards the development of a new class of effective HIV vaccines.

**Fig. 1:**
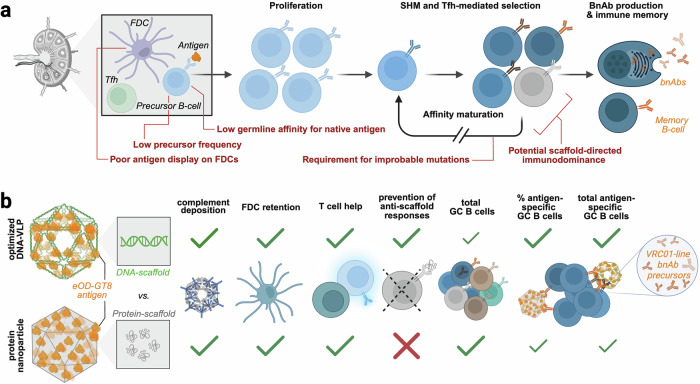
Immunogenic properties of DNA origami-based virus-like particles. **a** Schematic illustration of the challenges in generating broadly neutralizing antibodies (bnAbs) against HIV. Follicular dendritic cells (FDCs) in germinal centers (GCs) present the antigen (HIV Env) to rare precursor B cells with low affinity for the native antigen. Subsequent selection by T follicular helper (Tfh) cells and affinity maturation requires improbable mutations, restricting the development of bnAb-producing plasma cells and memory B cells. If conventional protein nanoparticle vaccines are used to present the antigen, immunogenicity of the protein scaffold adds additional challenges by diverting germinal center activity away from the antigen. **b** Schematic representation of the key findings of Romanov et al comparing DNA-origami-based virus-like particles (DNA-VLPs) and conventional protein nanoparticle vaccines, both displaying the germline-targeting HIV antigen eOD-GT8. Protein nanoparticle scaffolds can induce scaffold-directed immune responses, diverting GC activity toward anti-scaffold B cells. DNA-VLPs present the antigen while minimizing scaffold immunogenicity, resulting in higher relative and absolute numbers of antigen-specific GC B cells and successful generation of VRC01-lineage bnAb precursor B cells. Created in BioRender. Muecksch, F. (2026) https://BioRender.com/pk35waz

Despite decades of research, HIV remains without a licensed vaccine. Sterilizing protection will depend on potent and durable broadly neutralizing antibody (bnAb) responses capable of controlling the virus upon primary infection. The generation of bnAbs requires selection and affinity maturation of precursor B cells, which is uniquely challenging in the case of HIV due to a series of immunological and structural barriers^2^ (Fig. 1a). HIV-specific bnAb precursor B cells are exceedingly rare within the naïve repertoire, germline versions of these antibodies typically exhibit weak affinity for native Env, and recognition of conserved epitopes is limited through glycosylation and conformational masking. Even when appropriate B cell clones are recruited into germinal centers (GCs), affinity maturation towards neutralization breadth requires extensive and sometimes improbable somatic hypermutation.

Among conserved Env epitopes, the CD4 binding site (CD4bs) has emerged as a prime vaccine target, as CD4bs-specific bnAbs combine high potency and breadth with demonstrated protection in passive transfer studies in animals and clinical settings.^2,3^ VRC01-class bnAbs, derived from VH1-2 gene segments, mimic the interaction of the CD4 receptor with Env and achieve remarkable breadth and potency.^2^ However, Env-antibody interactions are sterically obstructed by N-glycans, and germline VRC01-class precursors bind native Env only weakly at best, necessitating carefully engineered germline-targeting immunogens to prime these lineages. Multivalent protein-based nanoparticle platforms have been developed to enhance B cell receptor (BCR) crosslinking and improve priming efficiency. Yet this strategy introduces an additional challenge: protein-based scaffolds can become immunodominant, eliciting robust antibody responses against the scaffold itself. Such off-target responses may compete with, or dilute GC reactions directed toward the intended epitope.

Romanov et al. hypothesize that presenting VRC01-targeting antigens on a DNA origami scaffold - built through precise nanoscale folding of DNA—could avoid anti-scaffold immune responses while enhancing expansion of antigen-specific B cells. To test this hypothesis, the authors employ previously characterized icosahedral DNA origami virus-like particles (DNA-VLPs)^4^ displaying eOD-GT8, an immunogen specifically engineered to bind germline precursors of VRC01-class bnAbs with high affinity.^5^ The folded DNA scaffold enabled antigen positioning across the three-dimensional surface, and DNA-VLPs presenting eOD-GT8 at distinct antigen density and valency were directly compared with an established protein-based nanoparticle platform presenting the same antigen^5^ for their ability to prime and expand VRC01-class precursor B cells.

DNA-VLP immunization of C57BL/6 mice induced antigen-specific antibody responses which, after boosting, markedly exceeded those elicited by monomeric eOD-GT8. Importantly, no detectable anti-DNA antibodies were generated by mice immunized with DNA-VLPs, confirming the low humoral immunogenicity of the DNA scaffold. However, a single dose of DNA-VLPs did not increase the number of total or antigen-specific GC B cells as well as T follicular helper (TFH) cells over those achieved by vaccination with the eOD-GT8 monomer. DNA-VLPs are sensitive to degradation by serum DNases but the limitation in GC responses was preserved in the absence of DNAse I, excluding particle stability as main efficacy limitation. In search of reasons for this weak priming, the authors observed limited follicular retention of DNA-VLPs. Increasing antigen density as well as overall valency on the DNA-VLPs enhanced follicular retention of DNA-VLPs. Consistent with the observed increased deposition of complement, this correlated with enhanced recruitment and expansion of antigen-specific B cells within GCs. Importantly, although vaccination with protein-based nanoparticles resulted in priming of overall larger total GC responses, the frequency of antigen-specific GC B cells elicited by the optimized DNA-VLPs was substantially higher, and the resulting total number of antigen-specific GC B cells did not differ between both vaccines.

Contrary to protein scaffolds, DNA-VLPs do not intrinsically provide T cell epitopes and T cell help must therefore be induced by the displayed protein antigen. As this lack of T cell epitopes might limit total GC B cell and TFH cell counts observed after DNA-VLP vaccination, the authors incorporated a synthetic universal CD4 T cell epitope (PADRE) into the immunogen design. Indeed, inclusion of PADRE enhanced total numbers of GC B cells and TFH cells, albeit to slightly lower levels than the protein-based vaccine. Importantly however, PADRE-inclusion slightly boosted the total number and strongly increased the frequency of antigen-specific GC B cells elicited by the DNA-VLP vaccine relative to the protein-based vaccine.

To directly assess priming of VRC01-class precursors, Romanov et al. next employed transgenic mice expressing the germline human IGHV1-2*02 gene segment within the endogenous murine Ig locus (VH1-2 mice), modeling frequency and diversity of potential bnAb precursor B cells of the B cell repertoire in humans. In this humanized system, optimized DNA-VLPs preferentially recruited and expanded antigen-specific B cells within GCs. This resulted in markedly higher frequencies of CD4bs-specific GC B cells relative to protein nanoparticle vaccination, which elicited a substantial amount of scaffold-directed antibody responses. BCR sequencing further revealed that vaccination with DNA-VLPs, but not with protein nanoparticles, effectively expanded CD4bs-specific GC B cell lineages showing typical signatures of VRC01 class antibodies.

Together, this study presents a prototype DNA origami scaffold with beneficial features to enhance HIV vaccine strategies by improving antigen-focused priming and expansion of rare precursor B cells without eliciting scaffold-directed antibody responses. Future studies will need to determine whether this early priming advantage—assessed primarily at day 14 and without boost immunizations in the humanized model—persists during sequential immunization and supports sustained affinity maturation and somatic hypermutation. Functional characterization of vaccine-elicited responses, including affinity and neutralization analyses of both polyclonal sera and isolated monoclonal antibodies, will be essential to establish whether precursor enrichment translates into bona fide bnAb activity. Finally, evaluation of long-term in vivo stability, manufacturability, and scalability of DNA-VLP platforms will be critical for clinical translation. DNA origami-protein immunogens emerge as novel and promising class of vaccines. The versatility and precision of DNA origami design offer enormous potential for further optimization of vaccine efficacy and breadth.
